# Comparison of a short version of the Food Frequency Questionnaire with its long version - a cross-sectional analysis in the Brazilian Longitudinal Study of Adult Health (ELSA-Brasil): Comparação da versão curta com a versão longa do Questionário de Frequência Alimentar - análise transversal no Estudo Longitudinal de Saúde do Adulto (ELSA-Brasil)

**DOI:** 10.1590/1516-3180.2014.00533004

**Published:** 2015-08-21

**Authors:** Livia Welter Mannato, Taisa Sabrina Silva Pereira, Gustavo Velasquez-Melendez, Letícia de Oliveira Cardoso, Isabela Martins Benseñor, Maria del Carmen Bisi Molina

**Affiliations:** I Msc. Postgraduate Student, Department of Public Health, Universidade Federal do Espírito Santo (UFES), Espírito Santo, Brazil.; II MSc. Doctoral Student of Public Health, Universidade Federal do Espírito Santo (UFES), Vitória, Espírito Santo, Brazil.; III PhD. Professor in the Department of Maternal and Child Nursing and Public Health, School of Nursing, Universidade Federal de Minas Gerais (UFMG), Belo Horizonte, Minas Gerais, Brazil.; IV PhD. Professor of the Postgraduate Program on Public Health Epidemiology, Fundação Oswaldo Cruz, ENSP/FIOCRUZ, Rio de Janeiro, Brazil.; V MD, PhD. Associate Professor, Department of Internal Medicine, School of Medicine, Universidade de São Paulo (USP), São Paulo, Brazil.; VI PhD. Associate Professor, Department of Integrated Health Education, Universidade Federal do Espírito Santo (UFES), Vitória, Espírito Santo, Brazil.

**Keywords:** Diet, Questionnaires, Validation studies, Epidemiologic studies, Nutrients., Dieta, Questionários, Estudos de Validação, Estudos epidemiológicos, Nutrientes.

## Abstract

**CONTEXT AND OBJECTIVE:**

The food frequency questionnaire (FFQ) is the preferred instrument for obtaining dietary information in epidemiological studies. A short form of the FFQ was compared with the original version that was used in the Brazilian Longitudinal Study of Adult Health (ELSA-Brasil), and also with three 24-hour dietary recalls.

**DESIGN AND SETTING:**

Cross-sectional study carried out in six Brazilian state capitals.

**METHODS:**

Multiple linear regression was used to reduce the original food and drink list of the FFQ, which had contained 114 food items. The frequency of consumption and nutritional composition of the foods were also taken into consideration. To assess the validity of the shortened FFQ, the energy and nutrients values of the 24-hour dietary recalls were deattenuated and log-transformed.

**RESULTS:**

The list of the FFQ of ELSA-Brasil was reduced to 76 food items. The intraclass correlation coefficients in the validation study ranged from 0.17 (selenium) to 0.66 (calcium).

**CONCLUSIONS:**

The number of items was reduced by 33%, while still maintaining relatively good capacity to measure energy and selected nutrients.

## RESUMO

**CONTEXTO E OBJETIVO:**

O Questionário de Frequência Alimentar (QFA) tem sido o instrumento de escolha para a obtenção de informações dietéticas em estudos epidemiológicos. Uma forma abreviada do questionário foi comparada com a versão original do QFA utilizada no Estudo Longitudinal de Saúde do Adulto (ELSA-Brasil), e também contra três registros alimentares (RA) de 24 horas.

**TIPO DE ESTUDO E LOCAL:**

Estudo transversal realizado em seis capitais brasileiras.

**MÉTODOS:**

Para redução da lista original de alimentos e bebidas do QFA, que contém 114 itens alimentares, foi empregada regressão linear múltipla, tendo sido consideradas também a frequência de consumo e a composição nutricional dos alimentos. Para avaliação da validade do QFA reduzido, os valores de energia e nutrientes dos registros alimentares de 24 horas foram "deatenuados" e "*log*-transformados".

**RESULTADOS:**

A lista do QFA ELSA-Brasil foi reduzida para 76 itens alimentares. No estudo de validade, os coeficientes de correlação intraclasse variaram de 0,17 (selênio) a 0,66 (cálcio).

**CONCLUSÃO:**

A redução dos itens foi de 33% e ainda assim foi possível manter a capacidade de medir relativamente bem energia e nutrientes selecionados.

## INTRODUCTION

The correlation between different levels of nutrient intake and occurrences of chronic diseases is very important for planning and implementing public health policies.^1^ ^2^ ^3^ ^and^ 4 One of the main challenges in nutritional epidemiology is to develop practical, valid and feasible methods for measuring diet.^5^ ^6^ ^and^ 7

Measuring food and nutrient consumption is not an easy task. This is due to the complexity of the human diet, and the intrinsic difficulties associated with methods used in dietary surveys.^5^ In this setting, food frequency questionnaires (FFQs) have been proven to be crucial for investigating the relationship between diet and health. 5 6 Additionally, FFQs make it possible to rank individuals according to consumption levels, and also allow links to be established between diet and specific events.^5^ ^6^ ^7^ ^and^ 8

Many FFQs have been developed for use in national epidemiological surveys, seeking to assess the usual diet of the population studied.^9^ Most of the questionnaires that include an extensive list of foods have higher non-response rates. The high number of food items in these questionnaires also increases the interview duration and the cost of the studies.^10^ In the Longitudinal Study of Adult Health (ELSA-Brasil), which was a prospective cohort study, a FFQ that included 114 items was specially created for the study^11^ in order to evaluate diet at the baseline examination. The mean time taken to apply the FFQ interview was 40 minutes. Given that the total duration of the ELSA-Brasil questionnaire was 4 to 6 hours, and the long interview was the most common complaint among the participants, it is very important to look for ways to shorten it.

## OBJECTIVE

The objective of this study was to construct a short FFQ based on the list of items included in the original version of the FFQ of ELSA-Brasil, in order to shorten the duration of the interview, but without any negative impact on the performance of the questionnaire. Our hypothesis was that it would be possible to shorten the questionnaire with no negative impact on performance through using the short version.

## METHODS

This study consisted of a reanalysis of the original validation study of the FFQ of ELSA-Brasil, focusing on decreasing the number of food items contained in the original FFQ, but without causing any significant alteration to its performance. The original FFQ of ELSA-Brasil, which contains 114 items, was validated in a sample consisting of 281 participants, of both sexes, aged 35-74 years, at six research centers in three regions of Brazil (southern, southeastern and northeastern regions). Further details on the individuals and how they were selected, and on the logistics of this study, which was conducted from October 2009 to October 2010, can be found in Molina et al.^12^

The original FFQ of ELSA-Brasil had three components: 1. foods/preparations; 2. measurement of portion intake; and 3. frequency of consumption, with eight response options ranging from "More than three times/day" to "never/almost never", and a column for participants to record their seasonal consumption of certain foods. To shorten the FFQ, some items were grouped together.^12^

### Data analysis

Estimates of nutrient intake were based on food compositions provided by the United States Department of Agriculture(USDA)^13^ and the Brazilian Table of Food Composition (TACO).^14^

The nutritional composition of regional preparations was calculated based on the individual components of each preparation, according to recipes from technical publications from educational and research institutions.^12^ For every 100 grams of edible portion of food and preparations, the following categories were calculated: total energy (kcal), carbohydrates (g), protein (g), fat (g), fiber (mg), calcium (mg), iron (mg), potassium (mg), selenium (mcg), zinc (mg), sodium (mg), vitamin A (IU), vitamin C (mg) and vitamin E (mg).

The distribution of consumption values for each nutrient was tested for normality of distribution using the Kolmogorov-Smirnov test. For variables that were not normally distributed, we applied logarithmic transformation.

### Short-form food frequency questionnaire 

To reduce the food list, the following procedures were performed, following the methodology published by Chiara et al. in 2007:^10^ 1. We estimated the Pearson correlations of the FFQ food items in relation to selected nutrients. From the correlation matrix, foods that had positive (r > 0.10) and significant (P < 0.05) correlation coefficients were selected for entry into the regression models. The per capita consumption of selected nutrients was considered to be a dependent variable, and the food items in the FFQ were taken to be an independent variable; 2. Linear regression models were estimated, adopting the stepwise method of inclusion of variables in the forward direction. The food items selected in the first stage were entered, so that the final model contained all items that helped to explain the use of the nutrients in question, regardless of the frequency of consumption; 3. Foods that, based on their nutritional composition, did not contribute to the explanation of the nutrient studied were excluded from the models; and 4. Beyond these first three steps, we also included foods in the short FFQ in situations in which they did not remain in the regression models but presented consumption percentages of 50% or more. 

### Relative validity analysis

To account for intra-individual variations in daily food intake, we obtained estimates of intra-individual and inter-individual variability from three 24-hour dietary recalls, as well as individual values for energy and nutrients, deattenuated according to intra-individual variability. The deattenuation process was performed using the method proposed by Iowa State University (ISU), using the PC-SIDEsoftware (software for intake distribution estimation for the Windows operating system), which had been developed by researchers in the statistics department of that university.^15^ Nutrients were adjusted according to total energy consumption, using the residual method proposed by Willett et al.^16^

We calculated mean values and standard deviations for the absolute values of energy intake, selected nutrients and these same nutrients adjusted according to total energy intake, obtained from the short-form FFQ and 24-hour dietary recalls. The Pearson correlation coefficient was used to compare the amounts of energy and nutrients from the short-form FFQ and the average from the three 24-hour dietary recalls. The acceptable correlation values between the two instruments ranged from 0.40 to 0.70.^16^ According to Nelson,^17^ intraclass correlation coefficient (ICC) values are smaller than Pearson correlation coefficients and, therefore, values greater than 0.4 show good agreement between the methods. We therefore calculated the ICC that evaluates the correlation between the information from the short-form FFQ and the average of the 24-hour dietary recalls.

To assess differences and possible distortions in the estimates of energy and nutrients obtained, between the methods (the short-form FFQ and the 24-hour dietary recalls), graphs showing the absolute differences between the values on the y-axis and the average intake calculated through the three 24-hour dietary recalls on the x-axis, and upper and lower limits of agreement (LOA) were constructed as proposed by Bland and Altman.^18^

## RESULTS 

Among the 281 study participants, 145 (51.6%) were female and 136 (48.4%) were male. Approximately 55% of the individuals were aged 35-54 years and 39% belonged to the functional educational category of technical level. Participation at each center ranged from 15.3% (Rio Grande do Sul; UFRGS) to 18.9% (Minas Gerais; UFMG).

From the Pearson correlation matrices, the nutrients were selected for each food, as follows: 82 foods that correlated with the total energy intake [r from 0.13 (grapes) to 0.36 (beans)]; 67 foods that correlated with carbohydrate intake [r from 0.12 (tea) to 0.41 (cassava)]; 42 foods that correlated with protein intake [r from 0.13 (beer) to 0.37 (beef without bone)]; 41 foods that correlated with lipid intake [r from 0.12 (crackers) to 0.42 (sausage)]; 27 foods that correlated with fiber [r from 0.12 (lentils) to 0.44 (oranges)]; 65 foods that correlated with potassium [r from 0.12 (strawberries) to 0.35 (cauliflower)]; 33 foods that correlated with selenium [r from 0.11 (cabbage) to 0.66 (walnuts)]; 15 foods that correlated with zinc [r from 0.14 (light bread) to 0.54 (boneless beef)]; 71 foods that correlated with sodium [r from 0.12 (polenta) to 0.38 (rice)]; 45 foods that correlated with vitamin A [r from 0.12 (liver) to 0.41 (carrot)]; 31 foods that correlated with vitamin C [r from 0.122 (guava) to 0.44 (mango)]; and 12 foods that correlated with vitamin E [r from 0.153 (nuts) to 0.38 (mango)]. The highest correlation coefficient was observed for the oil and selenium group (r = 0.65, P < 0.001) and the lowest coefficient was found for the leafy greens and selenium group (r = 0.11, P = 0.030).

Based on the correlation matrices, forward stepwise multiple linear regression was performed. The R^2^ ranged from 0.25 (vitamin E) to 0.83 (protein). The regression models resulted in 18 food items for energy (R^2^ = 0.62); 19 food items for carbohydrates (R^2^ = 0.72); 20 food items for protein (R^2^ = 0.83); 21 food items for lipids (R^2^ = 0.81); 8 food items for fiber (R^2^ = 0.57); 16 food items for calcium (R^2^ = 0.63); 9 food items for iron (R^2^ = 0.52); 15 food items for selenium (R^2^ = 0.71); 18 food items for sodium (R^2^ = 0.62); 13 food items for potassium (R^2^ = 0.54); 8 food items for zinc (R^2^ = 0.49); 16 food items for vitamin A (R^2^ = 0.63); 13 food items for vitamin C (R^2^ = 0.72); and 4 food items for vitamin E (R^2^ = 0.24).

The regressions resulted in 58 food items. Mayonnaise, which was derived from the regression of foods with potassium, was excluded because of the lack of plausibility and, thus, 57 food items remained. 

From the frequency list, we selected 15 food items that did not appear in the regressions, but which had consumption reports indicating frequencies greater than or equal to 50%. These were: zucchini/chayote/eggplant, garlic, crackers, coffee, manioc flour, lentils, watermelon, fried egg, boiled egg, cheese bread, polenta, pudding, okra, cabbage and grapes. The items of boiled egg and fried egg were incorporated as a single food item.

Subsequently, the researchers decided to incorporate wine and distilled spirits in the list. Since one of the objectives of ELSA-Brasil was to evaluate the effect of nutritional factors on the risks of developing obesity, diabetes and cardiovascular disease,^19^ we included these food items based on evidence from recent studies that have shown that moderate consumption of alcohol has a protective effect with regard to reducing the risk of noncommunicable diseases (NCD).^20^ ^21^ Besides these, the regional items "chimarrão" (green yerba mate tea) and "acarajé" (black-eyed pea fritters) were also included, since they showed high consumption among participants in their specific regions: 65% and 56%, respectively. Thus, the final version of the list of the ELSA-Brasil FFQ was reduced to 76 food items that explained 70% of the energy variability.

Table 1 presents the means and standard deviations for energy and nutrient intake for the original FFQ, shortened FFQ and 24-hour dietary recalls. The nutrient that showed the greatest variation with regard to reduction was sodium, both in the original and in the shortened FFQ. It was observed that after adjustments for energy, the average values of the nutrients decreased. 

**Table 1:**
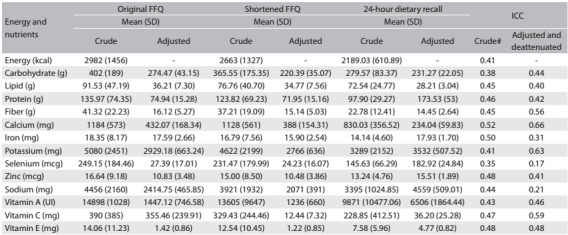
Mean and standard deviation (SD) for intake from original food frequency questionnaire (FFQ), shortened FFQ and 24 hour dietary recall, with intraclass correlation coeffient (ICC)

*Crude energy values. All correlations were statistically signifiant (P < 0.01)

The ICC ranged from 0.35 (selenium) to 0.52 (calcium) for raw nutrients, when adjusted for energy. Moreover, after deattenuation of the 24-hour dietary recall, the values for some nutrients were reduced and the ICC ranged from 0.17 (selenium) to 0.66 (calcium) (Table 1).

**Figure 1:**
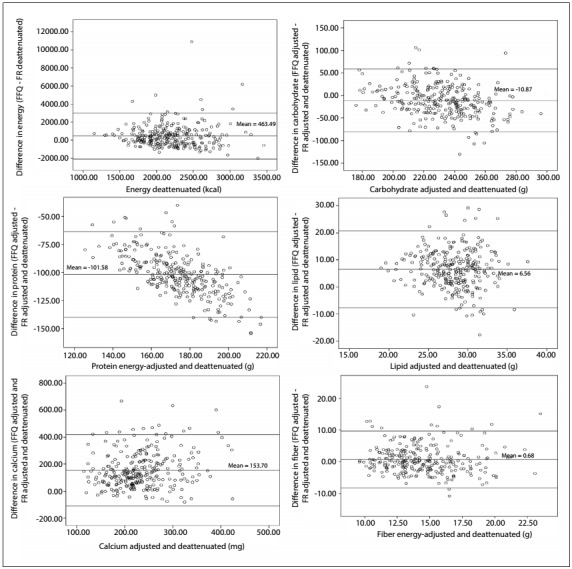
Scatter plots of the differences between the shortened food frequency questionnaire (FFQ) and food record (FR) methods.

Figure 1 shows the scatter plot of the differences between the shortened FFQ and 24-hour dietary recall methods, for energy and selected nutrients (carbohydrates, proteins, lipids, calcium and fiber). The average energy difference was 463.5 kcal (upper LOA = 3014.8 kcal; lower LOA = -2087.8 kcal); for carbohydrates, the average difference was -10.9 g (upper LOA = 59.0 g; lower LOA = -80.8 g); for protein, the average difference was -101.58 g (upper LOA = -63.4 g; lower LOA = -139.7 g); for lipids, the value found was 6.56 g (upper LOA = 20.8 g; lower LOA = -7.6 g); for calcium, it was 153.7 g (upper LOA = 417.1 g; lower LOA = -109.7 g); and for fiber, it was 0.68 g (upper LOA = 9.6 g; lower LOA = -8.3 g).

## DISCUSSION

To shorten the FFQ, methodology similar to that proposed by Chiara et al.^10^ was used, which enabled reduction of the original list of foods by approximately 66%. Among the 76 food items included, 58 came from the regression models, 14 from the list of 50% frequency reported by the participants, two from the researchers' decision (regional items) and two because they represented consumption by over 50% of the participants in their region. To validate this questionnaire, only the subset of the reduced food list was used, similarly to the technique used by Block et al.^21^

As found by Molina et al.,^11^ Henn et al.,^22^ Zanolla et al.,^23^ Giacomello et al.^24^ and Lima et al.,^25^ the average energy and nutrient intake was higher than the benchmark. This overestimation may be explained by the characteristics of the instrument, such as the perception of the portion consumed, memory and frequency of use that are established.^5^

When the nutrients were adjusted for energy, different behaviors were observed among the nutrients, because some correlation coefficients increased (protein, iron, potassium, selenium, zinc and sodium) and others decreased (carbohydrate, fat, fiber, calcium and vitamins A, C and E), as found by Crispim et al.,^26^ Zanolla et al.^23^ and Lima et al.^25^ According to Willett,^5^ the energy adjustment can increase the correlation coefficients when the variability of nutrient intake is related to energy intake, or it can decrease when the variability of the nutrient is subject to systematic errors of under or overestimation of reported food consumption.

Regarding the validity of the short-form FFQ, the values were similar to those reported in the literature. In the validation of the first FFQ developed for the Brazilian population, Sichieri and Everhart^27^ found correlation values ranging from 0.18 (vitamin A) to 0.55 (calcium). Giacomello et al.^24^ evaluated the performance of the FFQ developed by Sichieri and Everhart^27^ in a sample of pregnant women, and found correlation coefficients adjusted for energy ranging from 0.01 (unsaturated fat) to 0.47 (calcium). A study on the validity of a FFQ developed for a population of Japanese origin living in Brazil^28^ reported deattenuated and adjusted coefficients for the nutrients analyzed, which improved the correlation and increased the average coefficient from 0.47 to 0.56. Notably, the lowest coefficient found was for sodium.

In our study, the crude value of the intraclass correlation between the short-form FFQ and 24-hour dietary recalls ranged from 0.35 (selenium) to 0.52 (calcium). When the coefficients were adjusted for total energy intake and deattenuated according to intra-individual variability, different behavior was observed for the different nutrients studied. While increases in some values was observed, such as for carbohydrate, fiber, calcium, potassium and vitamins A and C, other nutrients such as lipid, protein, iron, selenium, zinc and sodium showed decreased values; only vitamin E remained the same. Overall, the coefficients ranged from 0.17 (selenium) to 0.66 (calcium).

In the study by Crispim et al.,^26^ after adjusting for total energy intake, it was found that the correlations for macronutrients decreased, while the correlations for micronutrients increased. As described by Zanolla et al.,^23^ it is important that the food frequency questionnaire should be able to correctly classify individuals according to intake levels, in order to obtain correct estimates of risk, which is essential for epidemiological studies. We also did an analysis on energy and five nutrients, using the method proposed by Bland and Altman.^18^ We observed that there was a reduction of the dispersion of the points for energy, carbohydrate, calcium and fiber when there was increased consumption of these nutrients. For proteins and lipids, there was an increase in the dispersion of the points, thus showing an increase in the differences between the dietary instruments. Our results for protein and carbohydrates showed low concordance between the short and the original questionnaire. Therefore, data on protein and carbohydrates have to be interpreted with some caution.

Some limitations should be taken into consideration in this study: the first relates to the intrinsic aspects of the methods used for diet evaluation. Itis possible that participants may list a single food item more than once, when it is included in more than one preparation, and thus overestimate the frequency of the food consumed. This could partially explain the overestimation using the short-form FFQ. Another limitation is the non-inclusion of biomarker testing in the validation process. Also, it should be noted that the reference method may not strictly constitute a gold standard measurement. The 24-hour dietary recall has to be considered to be the best option because it is less prone to recall bias and it does not depend on estimation of portion sizes, since participants use photographic models to estimate food portions. On the other hand, caution is required when using the short-form FFQ to evaluate nutritional deficiencies, since it could minimize the presence of these diseases.

## CONCLUSION

The short form of the ELSA-Brasil Food Frequency Questionnaire reduced the original 114 items to 76 food items. This version can be used in subsequent phases of the study, and in other similar studies, thereby enabling comparisons of nutrient consumption and making it possible to identify the relationship between diet, cardiovascular diseases and diabetes.
